# *Mycobacterium bovis* BCG infection of the brain following intravesical BCG treatment

**DOI:** 10.1128/asmcr.00186-25

**Published:** 2026-07-17

**Authors:** Sierra Amaya, Steven N. Schwartz, James E. Kirby, Hemant Varma

**Affiliations:** 1 Department of Pathology, Beth Israel Deaconess Medical Center and Harvard Medical School 376938, Boston, Massachusetts, USA; Pattern Bioscience, Austin, Texas, USA

**Keywords:** BCG, *Mycobacterium bovis*, brain abscess, CNS infection

## Abstract

**Background:**

Intravesical Bacille Calmette-Guerin (BCG) therapy with live-attenuated form of *Mycobacterium bovis* (*M. bovis*) is used as a treatment of early-stage bladder cancers. Documented adverse reactions are usually local and time limited, with severe central nervous system (CNS) complications being exceedingly rare. We report an 88-year-old patient with papillary urothelial carcinoma who presented with recurring neurological complications for over a year after treatment with intravesical BCG therapy.

**Case Summary:**

MRI at initial presentation revealed a dural-based left frontoparietal mass. Histological examination of the resected tissue revealed granulation tissue, necrosis, and lymphohistiocytic inflammation. Stains for organisms and cultures were negative, as was broad-range PCR. Two additional CNS resections performed followed detection of new lesions on follow-up. A mycobacterial culture from abscess fluid from the last resection recovered *M. tuberculosis* complex that, on region of detection signature pattern analysis, was identified as *M. bovis* BCG.

**Conclusion:**

This case represents one of the rare documented reports of an *M. bovis* BCG CNS infection resulting from intravesical BCG treatment. BCG infection should be considered in the differential of CNS infections in patients with a history of BCG treatment so early life-saving therapy can be instituted.

## INTRODUCTION

A connection between tuberculosis and anti-tumor activity was established in the 1920s (1). However, intravesical Bacille Calmette-Guerin (BCG) therapy for high-risk, non-muscle-invasive, early-stage bladder cancers has been in use since the 1970s. BCG is derived from an attenuated strain of *M. bovis* and is generally safe, with documented adverse reactions usually being local and time limited and severe central nervous system (CNS) complications being exceedingly rare (2, 3). Here, we report the case of a patient who developed CNS infection following intravesical BCG therapy.

## CASE PRESENTATION

**Fig 1:**
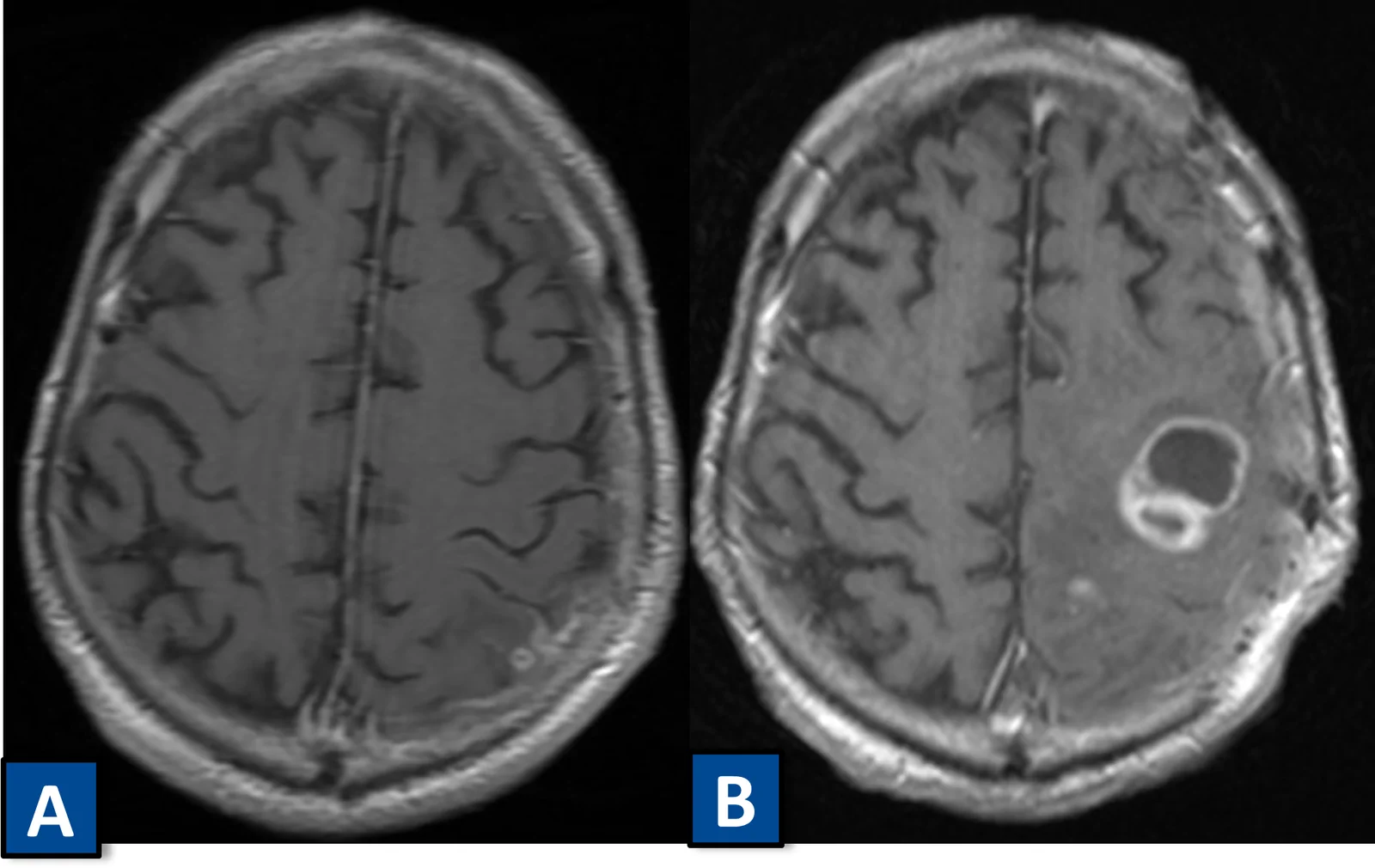
Post-contrast T1 axial imaging shows a dural-based left frontoparietal mass (**A**). Post-contrast T1 axial imaging shows an intraparenchymal enhancing mass (**B**).

The patient was an 88-year-old man originally from the United States with no known significant travel history, with suspected cerebral amyloid angiopathy, epilepsy, alpha-1 antitrypsin deficiency, hepatocellular carcinoma, and papillary urothelial carcinoma diagnosed in 2019. He underwent transurethral resection of his urothelial carcinoma, followed by six rounds of intravesical BCG therapy between February and May 2019. In May 2020, he presented to an outside hospital following a period of non-responsiveness and slurred speech. Magnetic resonance imaging (MRI) revealed a 3.1 cm enhancing dural-based left posterior frontoparietal mass with surrounding parenchymal vasogenic edema (Fig. 1A). The patient underwent craniotomy and surgical resection of the identified mass. Over the course of the following 18 months, two additional resections were performed following MRI detection of new lesions, the last after the patient developed seizures and right lower extremity weakness (Fig. 1B).

**Fig 2:**
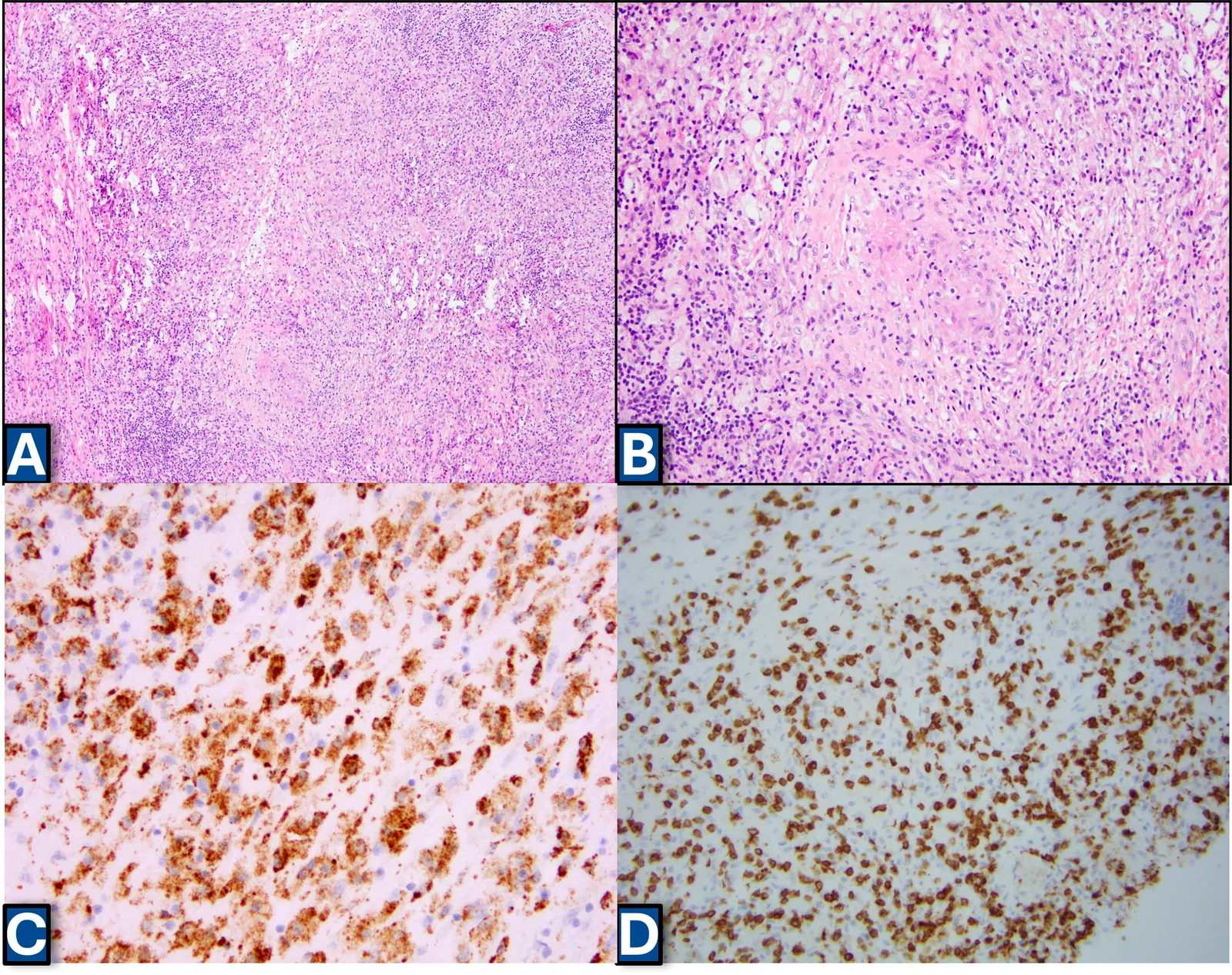
Hematoxylin and eosin stain shows dense inflammation, granulation tissue formation, and necrosis (**A**, 4× magnification; **B**, 10× magnification). Immunohistochemistry for CD68 shows a significant macrophage component to the inflammation (**C**, 20× magnification) and T-cell infiltrates (CD3 positive) (**D**, 20× magnification).

The resected brain tissue revealed granulation tissue, necrosis, and lymphohistiocytic inflammation with rare giant cells (Fig. 2A). No definitive necrotizing granulomas were seen; however, vague, poorly formed structures suggestive of granulomas were present (Fig. 2B). Immunohistochemical stains performed on the tissue demonstrated a T-cell predominant lymphocytic component highlighted by CD3 and macrophages highlighted by CD68 immunostains (Fig. 2C and D). Gram, Grocott methenamine silver (GMS), and acid-fast bacillus (AFB) stains performed on the resected tissue did not reveal any organisms. Resected tissue and swab cultures were negative for aerobic and anaerobic bacterial growth and fungal growth; no organisms were identified on AFB stains performed on abscess material. Broad-range polymerase chain reaction (PCR) performed at the University of Washington on the formalin-fixed paraffin-embedded tissue was similarly negative for bacteria, fungi, and tuberculous and non-tuberculous mycobacteria. A mycobacterial culture from abscess fluid obtained during the final resection resulted as positive for *M. tuberculosis* complex via matrix-assisted laser desorption/ionization time-of-flight mass spectrometry performed at the Massachusetts State Laboratory after the patient had been discharged to home hospice and 9 days before his death in December 2021. The sample was sent to the Centers for Disease Control and Prevention, where further testing of region of detection signature patterns resulted 1 month later identified *M. bovis* BCG.

## DISCUSSION

Cases of CNS infections due to BCG treatment are rare, and we identified six previously reported cases up to 2024; these cases, as well as the current case, are summarized in Table 1 (2, 4–8). All reported previous cases were male patients (age range 62–79 years, median 74 years) who presented with CNS symptoms between 6 months and 3 years following BCG treatment for a bladder neoplasm. Three cases involved the brain and/or meninges, while three cases presented as spinal epidural masses. Of note, only one of the previously reported cases identified *M. bovis* BCG organisms on AFB stain of tissue sections; in all other cases, the diagnosis was made via culture, often with subsequent molecular confirmation. One case was treated based on culture results from the bladder, with a presumptive diagnosis in the CNS rather than directly sampling the CNS tissue.

**TABLE 1:** Summary of previously reported BCG CNS infectious cases

Age (yr), sex	Diagnosis	Time from treatment to CNS presentation	Imaging	Histology	Microbiology	Treatment
88, male (9)	Papillary urothelial carcinoma	1 year	Dural-based frontoparietal mass, then enhancing parietal mass	Lymphohistiocytic inflammation, granulation tissue, necrosis	*M. tuberculosis* complex on culture, *M. bovis* confirmed via molecular testing	None
74, male (3)	Bladder cancer	<1 year	Cerebellar lesion with mass effect	Abscess	*M. bovis* on culture	Rifampin, isoniazid, pyrazinamide, ethambutol, steroids
73, male (2)	Transitional cell carcinoma	3 years	Nodular enhancement in basal ganglia and temporal lobe	Chronic granulomatous inflammation	*M. tuberculosis* complex on culture, *M. bovis* confirmed via PCR	Isoniazid, rifampin, ethambutol, pyrazinamide
79, male (4)	Grade 2 transitional cell carcinoma with stromal invasion	8 months	Osteomyelitis with anterior epidural abscess formation	None	*M. tuberculosis* complex on culture/PCR, *M. bovis* via whole-genome sequencing	Isoniazid, rifampin, ethambutol, pyridoxine, dexamethasone
64, male (5)	Urothelial vesical neoplasm	6 months	Thickening and linear meningeal enhancement	None	None (diagnosis presumed based on urine culture)	Isoniazid, rifampicin, ethambutol
75, male (6)	Grade 3 papillary urothelial carcinoma	2 years	Epidural abscess in spine, enhancing nodule in temporal lobe favored to be a tuberculous granuloma	None	*M. tuberculosis* complex on culture/DNA probe from epidural abscess aspiration	Isoniazid, rifampin, pyrazinamide, ethambutol, pyridoxine, dexamethasone
62, male (7)	Non-invasive bladder cancer	5–6 months	Enhancing mass lesion in spine with paraspinal extension	Granuloma, positive AFB stain	None	Isoniazid, rifampin, ethambutol

While our patient represents one of only a few cases of an *M. bovis* BCG brain abscess following BCG inoculation, proposed mechanisms of infection from other reports highlight possible routes of spread. Primarily, these include hematogenous spread, as this mechanism is thought to be responsible for the extragenitourinary complications of BCG therapy and *M. bovis* BCG dissemination in a mycobacterium-naïve patient (10). Microbial identification of *M. bovis* BCG organisms demonstrated here and in other reports suggests hematogenous dissemination of the organism from the bladder to the brain. Our patient underwent a transurethral resection of his bladder tumor prior to BCG therapy, which may have resulted in epithelial breakdown providing a potential entry point for *M. bovis* BCG to disseminate. The reason(s) for male predominance for the reported cases are not clear, though bladder carcinomas have a male predominance, and, as such, male patients are more likely to require BCG treatment (11). One possibility may be related to prostatic hypertrophy that makes males more susceptible to micro-trauma during catheter introduction for intravesical therapy. Alternatively, immune mechanisms may enhance male susceptibility to *M. bovis* BCG infection and brain infection, similar to the known increased male susceptibility to tuberculosis (12).

### Conclusion

All previously reported cases of *M. bovis* BCG infections in the CNS were successfully diagnosed and treated. However, there is likely selection bias, with undiagnosed cases not being reported. In our case, no organisms were detected on tissue sections or via broad-range PCR, and the etiology of the patient’s lesions remained unclear during his clinical course. Given the low clinical suspicion for a mycobacterial infection, empiric treatment was not initiated. Thus, BCG infections should be in the differential diagnosis of unusual abscesses and CNS infections, especially in male patients with a history of BCG treatment for bladder cancer, so that appropriate molecular testing and cultures can be used to confirm the diagnosis and early therapy can be initiated. Furthermore, as these presentations may occur months to years following BCG treatment, a careful review of patients’ histories and a low threshold of suspicion are warranted.

## Data Availability

Data relevant to the case report are included in the article.
